# New York Odontological Society

**Published:** 1888-03

**Authors:** 


### NEW YORK ODONTOLOGICAL SOCIETY.

REPORTED FOR THE INDEPENDENT PRACTITIONER.

A regular meeting of this Society was held Tuesday evening, February 14th, in the parlors of the New York Academy of Medicine. The President, Dr. J. Morgan Howe, occupied the chair.

Dr. S. G. Perry exhibited a light condenser for illuminating the mouth, in the form of a brass upright rod with right-angle arm, so constructed as to slide up and down the shaft and fastened at any point by a set screw. At the end of the right-angle or arm is attached a large lens, which by ingeniously contrived joints may be placed at any desired angle. The apparatus he uses is made as an attachment to the S. S. White chair, and is placed on the left side. It is arranged to swing clear of the patient's head. He finds it of great service on dark days in condensing rays of light for operations in the mouth; it also does excellent duty when used in connection with gas light. The apparatus may remain a fixture to the chair, or can easily be unscrewed from the arm if so desired. He has his condenser brought to him by his assistant every afternoon as soon as the light begins to fail, and fixes it to the chair.

Dr. Z. T. Sailer described his method of setting crowns. He lines a plate tooth with a gold backing, to which is soldered a gold pin or wire. The pin is split, or sawed through lengthwise. A platinum flanged tube or box is fitted to the root-canal and made secure by a filling. The split pin is warmed and coated with gutta-percha stopping, then pressed into the tube. Teeth engrafted in this manner are secure, and can be removed, if necessary, with no great difficulty.

Dr. J. Foster Flagg, of Philadelphia, gave to the meeting a lengthy and very interesting talk on "The Claims of Gutta-Percha as a Tooth-filling Material, with Methods of Manufacture, Testing, Heating and Manipulation." Before entering upon his subject he took occasion to make an exceedingly complimentary reference to the New York Odontological Society, which he considers the most prominent and influential local dental society in the country. Although not numbering so many resident members as do some others, he recognizes the fact that it is composed of gentlemen eminent in the profession, who have honored their calling and done much for its advancement. He felt it to be a great honor to himself to be invited to address so respectable a body.

Prof. Flagg then exhibited several pieces of crude gutta-percha as obtained from the manufacturers, and called the attention of the members to the great difference of seemingly like pieces. He offered for examination two of these pieces of equal thickness, one being considerably wider than the other, and requested the gentlemen inspecting them to compare the strips and give opinions regarding their flexibility, etc. Replies were given, that the narrowest piece appeared to be much stronger or tougher than the wider one. The reason for this difference, as explained by the doctor, is that even where several lots of gutta-percha were made from the same batch of gum and by the same process, the same results would not always follow. He was informed by the foreman of the works where he procured his crude gutta-percha, that when a quantity of gum was made up and rolled out there would appear several grades of the material. Some would be excellent, some not so good, and some exceedingly poor; consequently, great care should be exercised in selecting and preparing the gutta-percha for a tooth stopping. When fortunate enough to obtain a good quality of gutta-percha in any quantity, it should be kept in salt water, as the crude gum will deteriorate unless made into stoppings; but if thus made up it will remain without change for an indefinite period.

Samples of many preparations of gutta-percha were shown in the form of stoppings packed in glass vials, with carmine ink added, and all were found to leak. Dr. Flagg said that the pink baseplate gutta-percha, though exceedingly tough, leaked more than any of the other preparations. He thought it was not possible to make a filling of any form of gutta-percha that was proof against leakage. A friend had suggested varnishing the inside of the tube or vial with gutta-percha dissolved in chloroform, before introducing the filling. This he tried, and on being subjected to the same test it was found to leak in a few minutes. Stoppings were also carefully packed in little ivory cups, and after being tested with the ink were sawed in two, when the same results were observed. The products from the best manufacturers, although not absolutely proof against leakage, he thinks safe enough to protect the cavities from further decay.

A cavity in a large wooden tooth was filled by Dr. Flagg and passed around the room for inspection. It certainly presented a strong, hard filling. He prefers and uses serrated points for packing the gutta-percha, much like the large instruments used some years ago for condensing gold fillings. He objects to the practice so common with many operators of passing the stopping into or over the direct flame of the lamp, for it does not get uniformly heated in this way, the outside being over-heated while the centre is sometimes hardly warm. He instanced as an example a slice of bread held over a hot fire, which might be much scorched on the surface while the centre was not even crisp. No good or uniform filling can be made by this mode of heating. Just as much care is required in filling with gutta-percha as with gold. The rubber-dam should be adjusted to exclude any possibility of moisture, and the cavity carefully prepared or treated. Small bits of the stopping, after being properly heated, should be packed piece by piece with as much care as though using gold.

Replying to an inquiry as to his method of finishing gutta-percha fillings, Dr. Flagg stated that he used heated instruments for this purpose. He objected to the practice of wiping the stoppings with cotton saturated with chloroform, as it tended to soften the surface. He stated that many gutta-percha stoppings failed after being in the mouth for a time, from what he demonstrated "heat or mouth rot." In these cases the fillings seem to disintegrate.

Many dentists regard gutta-percha stoppings as mere temporary fillings, yet consider gold as permanent. He asked what proportion of cases were to be seen where gold fillings, after having been in the mouth three, five, or eight years, were not in such a condition that the point or edge of a burnisher could not be introduced around the margin. And when such fillings are removed, much tooth structure must be sacrificed before other fillings can be put in; gutta-percha fillings, though they may wear away somewhat, or "cup out," preserve the integrity of the tooth structure and can easily be replaced. These then should more justly be considered "permanent." Dr. Flagg being interrogated regarding copper amalgams, stated that where teeth were badly broken away or riddled with cavities and could be filled with nothing else, he thought copper amalgam would do good service.

On motion, the thanks of the society were tendered the essayist.

